# Supplementary material for: Point-of-care testing of hyponatremia and hypernatremia levels: An optoplasmonic biosensing approach
Source: PLoS One. 2025 Apr 23;20(4):e0319559. doi: 10.1371/journal.pone.0319559 (PMC12017493; doi:10.1371/journal.pone.0319559)
Supplement: Attachment — Submitted filename: Response to Reviewers.docx [file pone.0319559.s004.docx]

**Response to Reviewer’s Comments**

The authors would like to thank the reviewers for their valuable comments and suggestions and help us to improve this revised manuscript. Please find the point-by-point responses to the comments below, where relevant changes in the revised manuscript can be found in blue coloured texts. Reference numbers are kept as can be found in the revised manuscript (not re-numbered here) to avoid ambiguity.

**Reviewer #1**

Comments to Author

1. How does the imbalance of Na+ ions in blood or lymph impact a patient’s health and why is it crucial to monitor sodium ion (Na+) levels in the human body properly explain the medical cause?

We thank reviewer #1 for seeking more in-depth information on the medical causes and consequences of Na+ imbalance within human body. In the initial submission, we discussed how the off-standard presence of sodium ion in the regular body is detrimental to health and oftentimes related to other life-threatening diseases. In the revised version, we also added how the imbalance of sodium ion can be directly related to malfunctioning of kidney, metabolism and circulatory systems and any deficiency of immune system.

“The imbalance of bodily sodium can directly be associated to any malfunctioning of kidney, restricted operations of metabolism and circulatory systems and any deficiency of the overall immune system [10]. Any abrupt change of the Na+ level within mammals’ internally results in cellular swelling or shrinkage due to imbalanced osmotic pressure. Brain cells are heavily prone to this irregularity and can result in chronic subdural hemorrhage, permanent coma or even death in worst case scenarios [11]. Additionally, these patients typically have a high possibility of having cerebral edema (in hyponatremia) or vascular rupture (in hypernatremia) [12].”

References:

[10] “The Effect of Electrolytes on Blood Pressure: A Brief Summary of Meta-Analyses.” Accessed: Jan. 20, 2025. [Online]. Available: https://www.mdpi.com/2072-6643/11/6/1362

[11] R. M. Reynolds, P. L. Padfield, and J. R. Seckl, “Disorders of sodium balance,” BMJ, vol. 332, no. 7543, pp. 702–705, Mar. 2006, doi: 10.1136/bmj.332.7543.702.

[12] W. L. Wright, “Sodium and Fluid Management in Acute Brain Injury,” Curr. Neurol. Neurosci. Rep., vol. 12, no. 4, pp. 466–473, Aug. 2012, doi: 10.1007/s11910-012-0284-5.

2 What limitations in current point-of-care (POC) testing for Na+ levels does this study aim to address and do photonic crystal fiber (PCF)-based surface plasmon resonance (SPR) sensor detect those shortcomings?

We appreciate this question. We understand the conventional way of testing blood ion levels is the serum electrolyte test, which is not in situ since it involves sample centrifugation in the laboratory. Following that, the serum is placed into sample cups for examination on a fully automated clinical chemistry analyzer. Due to the delay between centrifugation and sample analysis, elevated false positive results are common findings in the laboratory [16]. For further validation, patients oftentimes need to undergo a number of other pricey procedures. Among point-of-care (POC) approaches, arterial blood gas analyzers and auto-analyzers proffered consistent results for the measurement of potassium ion whereas significant discrepancies are reported by Jain et. al. [19] in the case of sodium. The recent gas analyzer i-Smart 300E was able to demonstrate decent operation but in conjunction with a bulky setup associating disposable cartridge, reagents, waste bags, tubing, and sample probes [14]. These limitations of the existing POC setups are the primary motivation of this study to design a plasmonic sensor for this purpose.

In our investigation, the plasmonic sensor in question can **negate these shortcomings very efficiently**. This proof-of-concept work can be commercialized as a compact integrated kit. The arrangements discussed in figure 1 can be miniaturized into a simple handheld device with a diode laser, mini-spectrometer and a microcontroller where a sensing interface of a few millimeters is adequate for accurate detection. Therefore, our proposed biosensor can mitigate the aforementioned problems that arise while employing conventional devices for blood electrolyte analysis.

“Our proposed sensor surpassed these recently investigated biosensors in terms of sensing performance as well as has shown merit in outperforming typical bioassays for serum electrolyte concentration analysis. Being portable as an integrated POC kit and fast turnaround with high repeatability are the most promising aspects of our design.”

“Furthermore, this arrangement can be miniaturized into a simple and reusable handheld POC device with a diode laser, mini-spectrometer and a microcontroller since a sensing interface of a few millimeters is adequate for highly accurate measurements.”

References:

[14] B. Lee and H.-D. Park, “Performance evaluation of the i-Smart 300E cartridge for point-of-care electrolyte measurement in serum and plasma,” J. Clin. Lab. Anal., vol. 36, no. 4, p. e24295, 2022, doi: 10.1002/jcla.24295.

[16] A. Baruah, P. Goyal, S. Sinha, K. L. Ramesh, and R. Datta, “Delay in Specimen Processing-Major Source of Preanalytical Variation in Serum Electrolytes,” *J. Clin. Diagn. Res. JCDR*, vol. 8, no. 12, pp. CC01–CC03, Dec. 2014, doi: 10.7860/JCDR/2014/10150.5285.

[19] “Comparison of the point-of-care blood gas analyzer versus the laboratory auto-analyzer for the measurement of electrolytes | International Journal of Emergency Medicine.” Accessed: Jan. 20, 2025. [Online]. Available: https://link.springer.com/article/10.1007/s12245-009-0091-1.

3. How does the modified square lattice design improve the wave guidance and sensing performance of the proposed PCF sensor?

We appreciate reviewer’s interest in our novel semi-square lattice. Originally, a number of our coauthors first demonstrated (in 2019) the engineering of light-guidance efficiently by scaling down designated core airholes within a PCF for plasmonic sensing. One challenge while designing leaky microstrucuted fibers is to provide necessary mechanical strength to a structure. So, a thick layer of cladding is required. This restricts the feasibility of tailoring the leaked light energy out to the external surface to the SPP layer. In ref [28] and ref [29], it was proven that with a hexagonal lattice only, light can be strategically guided out to the outer plasmonic layer. However, this is the first time, we are showing this promising feature can be introduced in square shaped lattice arrangements as well.

“One common challenge with PCF-based plasmonic sensors is to meticulously design the leakage paths of the light energy to excite the SPPs when there are thick cladding layers. Scaling the core airholes down to optimal sizes enables unprecedented control over the magnitude and direction of this energy transfer [28]. Our POC-compatible biosensor is based on a diligently modified semi-square 2-ring lattice structure which ensures very good light-tailoring inside the PCF as we desired. This is the first demonstration of a propagation controlled core [29] being implemented with a square-shaped lattice by breaking the hexagonal periodicity.”

References:

[28] F. Haider, R. A. Aoni, R. Ahmed, W. J. Chew, and G. A. Mahdiraji, “Alphabetic-Core Assisted Microstructure Fiber Based Plasmonic Biosensor,” *Plasmonics*, vol. 15, no. 6, pp. 1949–1958, Dec. 2020, doi: 10.1007/s11468-020-01220-9.

[29] F. Haider, R. A. Aoni, R. Ahmed, Md. S. Islam, and A. E. Miroshnichenko, “Propagation Controlled Photonic Crystal Fiber-Based Plasmonic Sensor via Scaled-Down Approach,” *IEEE Sens. J.*, vol. 19, no. 3, pp. 962–969, Feb. 2019, doi: 10.1109/JSEN.2018.2880161.

4. Why was ITO chosen as the plasmonically active material, over other material like Au, Ag and how does it enhance the sensor's performance?

We welcome review’s question about the selection of our plasmonic layer. Indium Tin Oxide has been established as a conducting metal oxide with sharp resonances at near-IR frequencies. Around the telecommunication wavelengths, the material loss is minimized and thereby it is easy to ensure modal loss due to SPR is maximized. With ITO at the sensing interface, the operation window can be easily tuned unlike Au and Ag. Owing to Gold’s plasma frequency, its SPR peaks are usually limited under 1 micron. Moreover, ITO does not have the rapid oxidation problem which is typically encountered with silver.

“Unlike silver and gold, ITO does not suffer from oxidation problems in aqueous environments and exhibits an operational window around telecommunication wavelengths.”

References:

Dash, Jitendra Narayan, and Rajan Jha. "SPR biosensor based on polymer PCF coated with conducting metal oxide." IEEE Photonics Technology Letters 26.6 (2014): 595-598.

5. How does structural asymmetry-induced birefringence contribute to the superior sensing response in the y-polarized mode compared to the x-polarized mode?

Thanks for the interesting question. The asymmetry we introduced with the novel semi-square lattice opens 2 energy leaking routes opposite to each other as can be seen in fig 2. The orientation of them is vertical in our perspective view, which is contributing to a dominating y-pol. mode of the light. In addition to this, the asymmetry is also rendering a boosted birefringent response from our configuration. Thus, we concluded that the structural asymmetry-induced birefringence is the underlying cause of the suppressed x-pol response.

6. What does a sensitivity of 15,157 nm/RIU indicate about the sensor's performance or this is just a numerical figure only?

We thank reviewer #1 for seeking a detailed understanding. Wavelength sensitivity is the direct indicator of a sensor’s performance and is not a mere hypothetical number. In fig 3a, we can see the shift of the modal loss peak when the analyte RI is varied. The maximum sensitivity of 15,157 nm/RIU is also obtained from these data. The number is implying that an index change of unity at the surrounding region of the fiber sensor will result in a shift of 15,157 nm of the SPR peak respect to its previous spectral position.

7. What is the significance of achieving a detection resolution in the order of 10^−6^ RIU in practical applications?

Thanks for the query. Obtaining fine resolution is highly desirable in imaging, sensing and actuation applications as it indicates the resolvability of the smallest possible amount. Here, we obtained a detection resolution in the order of 10^−6^ RIU with our proposed POC-biosensor. This means that an RI change within the range of that small order can be successfully identified. This resolution is industry grade for biosensing applications and therefore, it definitely has practical significance.

8. How does the proposed sensor compare to conventional serum electrolyte panels currently used in medical diagnostics?

We appreciate the question. We discussed a comparison of our POC-compatible sensor with competing table-top setups like serum electrolyte panels and gas analyzers in our updated manuscript. The traditional devices are not in situ applicable since they involve sample centrifugation in the laboratory. Following that, the serum is placed into sample cups for examination on a fully automated clinical chemistry analyzer. Due to the delay between centrifugation and sample analysis, elevated false positive results are common findings in the laboratory [16].

References:

[16] A. Baruah, P. Goyal, S. Sinha, K. L. Ramesh, and R. Datta, “Delay in Specimen Processing-Major Source of Preanalytical Variation in Serum Electrolytes,” *J. Clin. Diagn. Res. JCDR*, vol. 8, no. 12, pp. CC01–CC03, Dec. 2014, doi: 10.7860/JCDR/2014/10150.5285.

9. In what ways could this sensor be utilized as a POC alternative for rapid Na+ ion testing?

We thank our reviewer for bringing up this issue. Basically, our proof-of-concept work can be commercialized as a compact integrated kit. The arrangements discussed in figure 1 can be miniaturized into a simple handheld device with a diode laser, mini-spectrometer and a microcontroller where a sensing interface of a few millimeters is adequate for accurate detection. Therefore, this can be employed even as patient’s personal level usable device. Hence, we claim our approach of dealing with the real-time Na+ measurement completely POC-compatible.

10. Update the introduction section of the article with some latest models of PCF SPR sensor like mereger of IMD and EMD shaped model, EMD and D shaped model, meger of spectroscopy and RI sensing etc. Cite them in the intoduction section?

https://doi.org/10.1007/s11468-023-01940-8

https://doi.org/10.1016/j.measurement.2021.110513

<https://doi.org/10.1016/j.ijleo.2022.169892>

We thank the reviewer for suggesting interesting reviewed literatures. We cited the references to strengthen our work.

**Reviewer #2**

Comments to Author

1. In the description under Figure 3, it is stated that “The sensor sensitivity peaks at test RI 1.3674 (concentration = 440 mg/dL) to a magnitude of 470 RIU-1 in the amplitude interrogation as visualized in the figure”. However, according to Figure 3(b), the amplitude sensitivity of 470 RIU-1 corresponds to an RI of 1.3810. Moreover, Table II lists the concentration of 440 mg/dL as correlating with an RI of 1.3874. Can the authors clarify this apparent discrepancy? It is crucial to ensure that the text accurately reflects the data presented in figures and tables.

We highly appreciate the careful reading of your manuscript by reviewer #2. We are extremely sorry to print a wrong value mistakenly. The analyte RI is 1.3810 (concentration = 380 mg/dL) and we have corrected that in the revised paper.

“The sensor sensitivity peaks at test RI 1.3810 (concentration = 380 mg/dL) to a magnitude of 470 RIU^-1^ in the amplitude interrogation as visualized in the figure.”

2. The manuscript states under Table II that “Initially, a polarizer followed by a broadband light source inputs the IR wavelengths into the fiber over a preassigned interval (typically a few seconds).” This sequence suggests that the polarizer is positioned before the light source, which is unconventional and possibly incorrect. Typically, the light source should precede the polarizer in the setup. Therefore, the correct phrasing should likely be, “A broadband light source followed by a polarizer inputs the IR wavelengths into the fiber…” Additionally, to ensure alignment with this setup, it is recommended to include a polarizer oriented in the Y-direction between the laser source and the single-mode fiber (SMF) in Figure 1(c). Could the authors revise the experimental setup accordingly to avoid potential confusion?

We apologize for the confusion and appreciate the reviewer’s help to identify the discrepancy. We have corrected the mistake and redrawn the figure 1 with a polarizer and further details.

“Initially, a broadband light source followed by a polarizer inputs the IR wavelengths into the fiber”

**Reviewer #3**

Comments to Author

The authors propose a photonic crystal fiber (PCF) sensor with a modified square lattice to achieve a propagation-controlled core, enhancing wave guidance and improving sensing performance. While the manuscript aligns with the journal's scope and presents intriguing findings, clarification of underlying mechanisms and additional references are required. Major revisions are recommended before acceptance.

We thank reviewer #3 for acknowledging our work and the effort behind it.

1. In the introduction, briefly elaborate on the novelty of this work and clarify the unique properties of the proposed SPR PCF sensor structures compared to other SPR sensor approaches (e.g., Nanomaterials 11(8), 2097 (2021); Micromachines 14(2), 340 (2023), Plasmonics 19 (1), 481-493 (2024) and DOI: <https://doi.org/10.1007/s11468-024-02319-z>). Include these references to strengthen the context.

Thanks to reviewer #3 for recommending the articles for our perusal. We discussed about the MIM structures and metamaterial absorbers in the revised version of the manuscript and cited the published works addressed in your comment.

2. Provide detailed settings for the COMSOL simulations, including the thickness of the perfectly matched layer (PML), scattering boundary conditions, and mesh size.

We appreciate reviewer #3 asking the details of the simulation parameters for increasing reproducibility. The cross-sectional area of the fiber is triangulated for the FEM calculations with the help of COMSOL’s built-in physics models. The final mesh consists of 15046 domain elements and 1348 boundary elements. Also, a PML with a thickness of 2 microns is found sufficient for absorbing outward scattering light. These information are included in the main body of the updated manuscript.

“we showed the FEM-based analysis results with COMSOL Multiphysics where the entire geometry is meshed into 15046 domain elements and 1348 boundary elements”

3. Specify the values of Sellmeier constants B1, B2, B3, C1, C2, and C3 in the manuscript.

We have added the values of the Sellmeier constants in the new version of our paper.

“Here, *n* is in the RI of fused silica and *λ* is the associated wavelength in the micrometer unit. Sellmeier constants are collected and plugged into the equation (1) from [41] where B_1_ = 0.69616300, B_2_ = 0.407942600, B_3_ = 0.897479400, C_1_ = 0.00467914826, C_2_ = 0.0135120631, C_3_ = 97.9340025.”

References:

[41] A D-Shaped Photonic Crystal Fiber Refractive Index Sensor Coated with Graphene and Zinc Oxide.” Accessed: Jan. 20, 2025. [Online]. Available: https://www.mdpi.com/1424-8220/21/1/71

4. Add relevant references on SPR PCF sensors, such as Crystals 13(5), 813 (2023) and Photonics 9(12), 916 (2022), to enhance the mechanism and background of SPR PCF sensors in context.

We are grateful to the reviewers for finding interest in our mechanism part. We have added the suggested references to discuss the principle of the sensing operation.

5. Define birefringence in the text and include references on birefringence in PCFs, such as Prog. Electromagn. Res. B 22, 39–52 (2010) and Jpn. J. Appl. 46(11L), L1048 (2007).

We defined birefringence clearly in the revised paper for better understanding of the readers.

6. Improve the quality of Figures 2(a) and (b) and include a color scale bar for better visualization.

Thanks for suggesting improvements to the figures. We have added a color scale bar in Fig. 2 to portray the normalized E-field intensity.

7. Add the simulation method and emphasize the potential applications of this work in the conclusion section.

We have mentioned that the simulation is done with FEM-method in the conclusion section and emphasized more on the prospective applications of our presented biosensor.

“In the FEM-based simulations, we validated the structural tolerances are within practical limits. We anticipate that the proposed polarization-sensitive sensor will be an effective POC tool for hypo- or hypernatremia fast identification in both at-home and nosocomial circumstances.”

8. For Fig. 3(a), explain the mechanism behind the significant change in confinement loss when nₐ varies from 1.3743 to 1.3810.

We figured that the continuous decline in RI contrast difference between the fundamental mode and the plasmon mode is the ultimate reason of the increasing confinement loss when nₐ varies from 1.3743 to 1.3810. And we incorporated this discussion into the main body of our edited manuscript.

“Here, it is noteworthy that the forward-shifting of resonance is also causing increased loss depths. This is because of a subtle continuous decline in RI contrast difference between the fundamental mode and the plasmon mode for escalated concentration of Na^+^ ions”

9. Additionally, clarify why nₐ = 1.3874 exhibits a larger FWHM compared to lower nₐ values.

Thanks a lot for raising this interesting question. By nature, ITO has greater penetration depth in the longer wavelengths. As the light is able to go beyond the thin film, we considered at our plasmonic layer, the SPR loses its steepness owing to the light density being broadband than other materials like Ag or Au. However, we found that our FWHMs are narrow enough to render practical sensing performance as elucidated in the modified manuscript during the revision phase.

References:

Dash, J. N., & Jha, R. (2016). Highly sensitive side-polished birefringent PCF-based SPR sensor in near IR. *Plasmonics*, *11*, 1505-1509.

10. Finally, specify the detection range of nₐ for the designed SPR PCF sensor.

The RI or sodium concentration sensing range of our proposed sensor is the full range listed in Table II so that it can execute detection of any level of the Na+ electrolyte imbalance.
