# Supplementary material for: Point-of-care testing of hyponatremia and hypernatremia levels: An optoplasmonic biosensing approach
Source: PLoS One. 2025 Apr 23;20(4):e0319559. doi: 10.1371/journal.pone.0319559 (PMC12017493; doi:10.1371/journal.pone.0319559)
Supplement: Attachment — Submitted filename: pone.0319559.doc [file pone.0319559.s005.doc]

^[1]^

^1^The Wyant College of Optical Sciences, The University of Arizona, Tucson, AZ, 85721, USA.

# Point-of-care testing of hyponatremia and hypernatremia levels: an optoplasmonic biosensing approach

Abdullah Mohammad Tanvirul Hoque^1,^ ^¶^, Abrar Islam^2,3,^ ^¶,^*, Firoz Haider^4^, Rifat Ahmmed Aoni^5^, and Rajib Ahmed^6,7^

^2^Department of Electrical and Electronic Engineering, Bangabandhu Sheikh Mujibur Rahman Science and Technology University, Gopalganj 8100, Bangladesh.

^3^Department of Electrical Engineering, The Pennsylvania State University, University Park, PA 16802, USA.

^4^Faculty of Engineering, The University of New South Wales, Sydney, NSW 2052, Australia.

^5^The Mork Family Department of Chemical Engineering and Materials Science, University of Southern California, Los Angeles, CA 90089, USA.

^6^School of Medicine, Stanford University, Palo Alto, CA 94304 USA.

^7^School of Bioengineering and Health, Wuhan Textile University, Wuhan, Hubei, 430079, China.

*Corresponding author

Email: [abrar@psu.edu](mailto:abrar@psu.edu)

^¶^ These authors contributed equally to this work.

##### **Abstract**

In the human body, sodium ion (Na^+^) is the principal extracellular cation and its imbalance in blood or lymph results in either hyponatremia or hypernatremia conditions. Any of these scenarios have fatal effects on a patient’s health over time. However, the existing technologies limit quick point-of-care (POC) testing options. To overcome the challenges associated with prompt diagnosis, we proposed a photonic crystal fiber (PCF) based surface plasmon resonance (SPR) sensor for in situ rapid testing of Na^+^ ion levels in patient’s blood samples. A unique PCF sensor with a modified square lattice is engineered to achieve a propagation-controlled core that permits enhanced control over wave guidance resulting in improved sensing performance. Plasmonically active conducting indium tin oxide (ITO) is used to omit the limitations that arise from undesired oxidation with time and to enable higher spectral tunability. The finite element method (FEM) is used to perform the numerical analysis on performance and all sensor parameters are optimized to ensure the best sensing outcome. The structural asymmetry-induced birefringence enforced a superior y-polarized sensing response compared to that of the x-polarized mode for the proposed sensor, where it proffers the highest wavelength sensitivity of 15,157 nm/RIU with a detection resolution of 6.61 × 10^−6^ RIU. Additionally, the sensor exhibits a maximum amplitude sensitivity of 470 RIU^-1^ and an outstanding figure of merit (FOM) of 171 RIU^-1^. Due to the high sensitivity and resolution, we infer that the proposed sensor will be a promising POC alternative to the conventional serum electrolyte panels used in present medical practice.

*Index Terms*—Biosensor, Surface plasmon resonance, Hyponatremia, Hypernatremia, Finite element method (FEM), Photonic crystal fiber, Sensitivity, FOM.

##### **Introduction**

INSIDE a human blood circulatory system, sodium ion (Na^+^) is one of the most crucial electrolytes of extracellular fluid which is responsible for the generation of action potentials in nervous and cardiovascular tissues [1]. This makes the balanced presence of Na^+^ an imminent requirement for the body to function properly. Although our body possesses a sophisticated mechanism for maintaining the optimal amount of electrolytes within our bloodstream, reported cases of unstable sodium levels are very frequent in patients with kidney problems, different viral & microbial infections, diarrhea & diabetes mellitus [2], [3]. Cancer patients are sometimes required to be under constant observation for abnormal serum sodium level [4]. Repetitive encounters of out-of-balance electrolytes in bodily fluid are found in COVID-19 patients which led to sodium concentration in blood being an indirect prognostic marker of the disease [5]. Among patients admitted to hospitals, individuals with electrolyte imbalance had a greater mortality ratio than the rest [6]. Lately, Lo et. al. [7] suggested dysnatremia or extreme abnormality of plasma Na^+^ level is the highest reported electrolyte disorder in patients with liver cirrhosis and heart failure.

Since normal serum Na^+^ concentration is mostly recognized within the range of 135 to 145 mmol/L (135 to 145 mEq/L), hyponatremia (i.e., low blood sodium level) is defined as a concentration of sodium being less than 135 mmol/L [8]. On the other hand, hypernatremia is typically characterized as a serum sodium level greater than 145 mmol/L [9]. The imbalance of bodily sodium can directly be associated to any malfunctioning of kidney, restricted operations of metabolism and circulatory systems and deficiencies of the overall immune system [10]. An abrupt change of the Na^+^ level within mammals’ internally results in cellular swelling or shrinkage due to imbalanced osmotic pressure. Brain cells are heavily prone to this irregularity and can result in chronic subdural hemorrhage, permanent coma or even death in worst case scenarios [11]. Additionally, these patients typically have a high possibility of having cerebral edema (in hyponatremia) or vascular rupture (in hypernatremia) [12]. Even a mild Na^+^ ion imbalance typically causes dizziness, brain swelling, headaches, nausea, excessive blood pressure, and sudden loss of stamina [13]. Moreover, electrolyte measurements are important in the differential diagnosis of a variety of illnesses, including diabetic ketoacidosis and renal tubular acidosis [14]. Rigorous physical exams and patient history are necessary to help identify the core reasons for the sodium imbalance and to administer any treatments [8]. In certain situations like individuals with persistent hyperosmolality, intensive treatment with hypotonic fluids may be required immediately [15].

The conventional way of testing blood ion levels is the serum electrolyte test, which is not in situ since it involves sample centrifugation in the laboratory. Following that, the serum is placed into sample cups for examination on a fully automated clinical chemistry analyzer. Due to the delay between centrifugation and sample analysis, elevated false positive results are common findings in the laboratory [16]. For further validation, patients oftentimes need to undergo a number of pricey procedures, including liver function tests, computed tomography (CT) scans, and electrocardiography [17], [18]. Among point-of-care (POC) approaches, arterial blood gas analyzers and auto-analyzers proffered consistent results for the measurement of potassium ion, whereas significant discrepancies are reported by Jain *et. al.* [19] in the case of sodium. A recent gas analyzer i-Smart 300E was able to demonstrate decent operation but in conjunction with a bulky setup involving disposable cartridge, reagents, waste bags, tubing, and sample probes [14]. Therefore, a fast and affordable compact POC testing system of blood electrolytes is critically needed in current medical practice to expedite the diagnosis without compromising accuracy and repeatability.

Here, we propose a novel bio-optical method for the real-time measurement and monitoring of Na^+^ levels in serum, which can be employable at both medical and patient’s home settings. In this work, we leveraged the change in optical properties (refractive index) of serum due to any minute variation in the sodium ion concentration in blood and engineered a lossy waveguide (photonic crystal fiber or PCF) to quantify the change. The amplitude of the loss in optical waveguides and the spectral position of the loss peaks are very susceptible to the refractive index of surrounding materials when the whole structure is plasmonically active. The plasmonic microstructure is easy to obtain as any conductor-dielectric interface is physically bound to generate surface plasmon polariton (SPP) under an applied electric field through electromagnetic interactions. The energy required for the oscillation of SPPs is collected from the applied light into the waveguide rendering the propagation loss in principle. When the frequency of the transmitted electromagnetic wave matches the free electron oscillation frequency, the loss accentuates which is defined as the surface plasmon resonance (SPR). That implies, at resonance, the confinement of light inside the waveguide is minimal and the loss profile at this resonant wavelength is the basis of our measurement scheme.

Using the SPR phenomena, refractive index (RI) sensors are positively advocated by researchers and sensor technologists because of their highly sensitive nature, design flexibility, and compactness [20], [21]. To leverage these favorable features, metal-insulator-metal structures and meta-absorber systems are being investigated recently [22], [23], [24]. However, the popular choices of SPR sensor platforms are still either prism-based or fiber-based. Prism-based sensors were a traditional approach in SPR technologies, which are large and heavy in design since optomechanical rotary parts are involved [25]. On the other hand, miniaturization and portability are attained when metal-coated optical fiber is used instead of prisms. However, precise control over the doping concentrations in graded-index fibers and the jacket removal procedure make the fabrication of conventional fiber-based sensors relatively complicated. These reasons favor PCF as the leaky waveguide over other candidates for sensor applications, since controlled confinement of the propagating light and SPP excitation can be done very conveniently.

Here, a novel PCF-based SPR sensor is designed and critically investigated in terms of realization feasibility and sensing performance as an in-situ blood Na^+^ testing kit. There are reported numerical studies on SPR sensors for label-free general-purpose refractometry of different chemical and biomolecular solutions with moderate sensitivities [26]. For example, the highest wavelength sensitivity of a proposed liquid sensor went up to 10,000 nm/RIU using a titanium nitride-coated fiber [27]. More recently, plasmonic sensors targeting specific bio-analytes received higher attention by researchers owing to the drive of medical instrumentation industries. In 2022, a biosensor by Rajeswari and Revathi [28] yielded a maximum sensitivity of 6701.03 nm/RIU aiming for early screening of cancerous cells. Another machine learning-assisted SPR-based malaria sensor exhibited a sensitivity of 12,142 nm/RIU, 9736 nm/RIU and 8241 nm/RIU for ring, trophozoite and schizont stages of malaria parasite reproduction, respectively [29]. Lately, a photonic COVID sensor proffered a spectral sensitivity of 3948 nm/RIU [30]. In 2024, a hemoglobin sensor developed by Dai *et. al.* [31] was able to achieve wavelength sensitivities up to 7500 nm/RIU. Our proposed sensor surpassed these recently investigated biosensors in terms of sensing performance as well as has shown merit in outperforming typical bioassays for serum electrolyte concentration analysis. Transportability as an integrated POC kit and fast turnaround with high repeatability are the most promising aspects of our design [32]. In addition, this study explored a completely new avenue for estimating bodily present Na^+^ concentration in POC scenarios utilizing biophotonic engineering. Moreover, our finite element method (FEM)-based numerical analysis validates that the sensor has good fabrication tolerances of structural parameters to achieve the best possible sensing operation.

##### **Sensor design and theory**

In the sensor, a thin film of indium tin oxide (ITO) is used as a plasmonic layer due to its enhanced freedom of optical tunability by regulating the oxygen content. Unlike silver and gold, ITO does not suffer from oxidation problems in aqueous environments and exhibits an operational window around telecommunication wavelengths. One common challenge with PCF-based plasmonic sensors is to meticulously design the leakage paths of the light energy to excite the SPPs when there are thick cladding layers. Scaling the core airholes down to optimal sizes enables unprecedented control over the magnitude and direction of this energy transfer [33]. Our POC-compatible biosensor is based on a diligently modified semi-square 2-ring lattice structure which ensures very good light-tailoring inside the PCF as we desired. This is the first demonstration of a propagation controlled core [34] being implemented with a square-shaped lattice by breaking the hexagonal periodicity. Additionally, instead of drilling, this sort of fiber structure is easy to fabricate by stacking commercially available micro-capillaries and drawing the preform [35]. The four-fold asymmetry of the lattice mandates polarization dependency for our sensor resulting in a highly birefringent propagation profile. Because of the high flexibility offered by available PCF manufacturing technology, the plausible ways of designing SPR sensors are vastly exploited [26]. However, internal infusion of analyte into small capillaries is frequently criticized for posing uncontrollable loss [36]. Some reported works studying these sorts of SPR sensor configurations can be found in ref [37], [38], [39]. In addition, surface roughness arisen from fiber polishing is highly undesirable for any kind of actuation application. For these reasons, we adopted an external sensing approach employing an unpolished ITO-coated PCF. This means the serum sample can be glided onto the functionalized sensitive interface of the device from outside, which helps fast and complication-free cleaning and reusing.

**Fig. 1. (a) Cross-section of the proposed sensor, (b) Stacked PCF preform, (c) Schematic diagram of the experimental setup.**

Fig. 1(a) and 1(b) depict the cross-sectional view of the sensor and the stacked capillary arrangement of the vertically elongated core PCF, respectively. The air-holes radius at the core of the fiber is scaled down to regulate the light confinement and open energy leakage paths opposite to each other. Four solid glass rods are inserted adjacent to the center to stabilize the stacked microstructure (see Fig. 1(b)). This configuration allows better SPP excitation by rerouting the evanescent electric fields to four specific locations which resulted in increased analyte-ligand interactions. In this design, fused silica is used as the fiber material, and large air holes are introduced as the cladding so that total internal reflection limits the complete dissipation of the guided photon energy. Sellmeier equation is used to model the optical index of the background silica for the infrared (IR) band [40];

$n^{2}\left(\lambda\right)=1+\frac{B_{1}\lambda^{2}}{\lambda^{2}-C_{1}}+\frac{B_{2}\lambda^{2}}{\lambda^{2}-C_{2}}+\frac{B_{3}\lambda^{2}}{\lambda^{2}-C_{3}}$ (1)

Here, *n* is in the RI of fused silica and *λ* is the associated wavelength in the micrometer unit. Sellmeier constants are collected and plugged into the equation (1) from [41] where B_1_ = 0.69616300, B_2_ = 0.407942600, B_3_ = 0.897479400, C_1_ = 0.00467914826, C_2_ = 0.0135120631, C_3_ = 97.9340025. On top of the PCF, the conducting ITO layer is placed which can be deposited through either atomic layer deposition or any other nanoparticle deposition technique. For simulation purposes, we used the empirical Drude model below to approximate the complex dielectric constant of ITO [42]:

$\varepsilon(\omega)=\varepsilon-\frac{\omega_{p}^{2}}{\omega^{2}+j\omega\Gamma}$ (2)

Where, the intra-band dielectric constant, Ɛ = 3.964; damping coefficient, Г= 0.111eV; plasma frequency, $\omega_{p}^{2}=\left(\frac{ne^{2}}{\mu\varepsilon_{0}}\right)^{\frac{1}{2}}$= 2.19eV; provided that μ = 0.3 times free electron mass. To apply boundary conditions to our 2D cross-section, a cylindrical perfectly matched layer (2 µm) is employed for absorbing the scattered lights. Detailed optimization of dimensional parameters and computation of nanofabrication tolerances are articulated in the following section of this paper. Nevertheless, the optimal values are listed in the table below.

**TABLE I**

**Optimized sensor p**arameters

| Parameters | Value |
| --- | --- |
| Pitch size of PCF, Ʌ* | 2.2 µm |
| Cladding airhole diameter, d | 1.43 µm |
| Core airhole diameter, dc | 0.44 µm |
| Thin ITO layer thickness | 50 nm |

*Pitch size (Ʌ) is defined as the center-to-center spatial separation between two adjacent air holes.

To make our sensing scheme operational, we interpreted the molar density of serum sodium in terms of its RI and realized the immobilization of Na^+^ ion at the sensor surface. At room temperature, the plasma sodium RI can be correctly approximated by equation (3), where *C* denotes concentration in mgdL^−1^, and *k* indicates the concentration factor [43];

$n_{Na^{+}}=1.3373+1.768\times10^{-3}\left(\frac{C*k}{393}\right)-5.8\times10^{-6}\left(\frac{C*k}{393}\right)^{2}$ (3)

Table II summarizes several values defined by the relation given above and shows how different diagnostic decisions can be made based on extracted data from our proposed system. We assumed a midpoint (139.1 mmol/L or 320 mg/dL) of the prescribed normal sodium concentration range to be the Na^+^ level in a healthy body.

**TABLE II**

The RI depicted by patient’s plasma for different sodium levels

| Analyte | Concentration | | RI | Remarks |
| --- | --- | --- | --- | --- |
|  | mmolL^−1^ mgdL^−1^ | |  |  |
| Na^+^, *k*=30 | 86.95 | 200 | 1.3602 | Extreme hypo. |
|  | 113.00 | 260 | 1.3674 | Hyponatremia |
|  | 139.10 | 320 | 1.3743 | Normal |
|  | 165.20 | 380 | 1.3810 | Hypernatremia |
|  | 191.30 | 440 | 1.3874 | Extreme hyper. |

*The operation & remarks are not limited to these 5 concentrations only. Experimentation with these 5 concentration levels is done as the proof-of-concept. The sensor is operational for all RIs ranging 1.33-1.40.

Carboxylate or sulfonate bifunctional ligand and calcium silicate hydrate gel pores can be alternately used for immobilizing Na^+^ atoms over our predesignated plasmonic surface [44], [45]. Then, the attached ions will correspond to a certain RI for the analyte layer and modulate the resonant wavelengths of our fiber sensor. Fig. 1(c) depicts the functional schematic of our biosensing setup. Initially, a broadband light source followed by a polarizer inputs the IR wavelengths into the fiber over a preassigned time interval (typically a few seconds). Due to the leaky waveguide design, the SPPs bonded with sodium particles through ligands receive energy from the propagating light. Therefore, distinguishable confinement loss peaks appear when SPR occurs at specific wavelengths for individual sodium plasma samples if the concentration (or RI) is different in each case. The scanned spectral data is fed into a computer after being collected by an optical spectra analyzer or charged coupled device. Lastly, a quick post-processing and a comparison with modeled reference loss profiles give out the final results. Furthermore, this arrangement can be miniaturized into a simple and reusable handheld POC device with a diode laser, mini-spectrometer and a microcontroller since a sensing interface of a few millimeters is adequate for highly accurate measurements.

##### **Results & discussions**

To justify our proposed detection model, we showed the FEM-based analysis results with COMSOL Multiphysics, where the entire geometry is meshed into 15046 domain elements. Fig. 2 shows the electric field distribution throughout the whole cross-sectional area of this sensor at normal sodium level for y-polarized light input under fulfilled resonance conditions. Our polarization-dependent geometry resulted in a prevalent y-polarization excitation response in reference to the x-polarization mode operation. Electric field intensity profiles in fundamental core-guided mode (Fig. 2(a)) and SPP mode (Fig. 2(b)) indicate strong coupling between them. In addition, the energy leakage pathways originated from our engineered propagation-controlled core are revealed from the intensity color map. The phase matching (at resonance, real RI of core mode = real RI of SPP mode) of the aforementioned fiber modes is shown in Fig. 2(c) along with the sharp modal loss that appeared at the resonant wavelength of 1677 nm. The fiber propagation loss is evaluated as follows [46];

$\alpha\left(\frac{dB}{cm}\right)=8.686\times\frac{2\pi}{\lambda(\mu m)}I_{m}(n_{eff})\times10^{4}$ (4)

Here, *λ* = applied wavelength & *I_m_* = extinction coefficient of the modal effective index. When a light guiding system responds differently for two orthogonally linear polarized lights, the difference in their real RIs is defined as the birefringence profile of that system. The birefringence spectrum of the proposed photonic sensor for analyte RI 1.3743 is shown in Fig. 2(d). We can see intensified birefringent behaviors (up to 11.4×10^−4^ RIU) of the structure in longer wavelengths owing to increased light penetration depth at that region [47]. Any asymmetrical structure has a natural tendency to exhibit strong birefringence which enables better control of the polarization states of the input signal and increases the stability of the optical device [30].

We can see, both resonant wavelength and peak loss magnitude vary depending on the change in analyte layer RI. This is because, due to variations in analyte concentration, the net number of sodium ions varies and attaches to the carboxylate/sulfonate ligands accordingly. The number of active ligand-Na^+^ conjugates is dictated by analyte RI and thus, the required energy changes for satisfied SPR conditions for different analyte samples. The shifts in resonant wavelengths could be blue or red with an increase of ion concentration based on the type of PCF used. For our particular case, we observed redshifts of SPRs as the sodium level goes up in the patient’s blood stream. Fig. 3(a) portrays this fact in our working wavelength region for serum sodium concentrations tabulated in Table II. The molar concentration of 86.95 mmol/L depicts the lowest loss of 171 dB/cm at 1599 nm, whereas the highest loss of 444 dB/cm is evident for 119.3 mmol/L Na^+^ level at 1826 nm. All SPR data points of interest from those loss curves are summarized in Table III. Here, it is noteworthy that the forward-shifting of resonance is also causing increased loss depths. This is because of a subtle continuous decline in RI contrast difference between the fundamental mode and the plasmon mode for escalated concentration of Na^+^ ions [48].

**Fig. 2. (a) Electric field profiles of the core-guided mode and (b) SPP mode at analyte RI 1.3743. (c) Satisfied phase-matching condition, and (d) Birefringence behavior.**

The SPR wavelength shift per unit RI variation of the analyte is specified as the wavelength or spectral sensitivity (*S_λ_(nm/RIU) = ∆λ_res_/∆RI_analyte_*). The deeper light penetration into ITO as the working wavelength pushes towards mid-IR results in improved sensitivity in spectral interrogation. The highest wavelength sensitivity proffered by our presented biosensor is 15,157 nm/RIU at RI 1.3810 and the lowest magnitude is found 5,000 nm/RIU at RI 1.3602. Instead of analyzing the whole effective wavelength range, the amplitude-based investigation method for sensitivity characterization is also used here for challenging our capability under different detection approaches. Amplitude sensitivity is mathematically addressed as follows [49],

$S_{A}\left[RIU^{-1}\right]=-\frac{1}{\alpha(\lambda,RI_{analyte})}\frac{\partial\alpha(\lambda,RI_{analyte})}{\lambda,RI_{analyte}}$ (5)

**TABLE III**

Performance analysis for the proposed PCF sensor in the detection of Hyponatremia and Hypernatremia

| Analyte RI | Res. Wave. (nm) | Peak Loss (dB/cm) | Res. Shift (nm) | Wave. Sens. (nm/RIU) | Ampl. Sens. (RIU^-1^) | FWHM (nm) | FOM (RIU^-1^) |  | Wave. Res. (RIU) | SNR | DL (RIU^2^/nm) |
| --- | --- | --- | --- | --- | --- | --- | --- | --- | --- | --- | --- |
| 1.3602 | 1599 | 171 | 36 | 5000 | -58 | 196 | 26 |  | 2.00×10^-5^ | 0.184 | 1.32×10^-9^ |
| 1.3674 | 1635 | 203 | 42 | 6087 | -99 | 167 | 37 |  | 1.64×10^-5^ | 0.251 | 1.08×10^-9^ |
| 1.3743 | 1677 | 257 | 52 | 7762 | -205 | 143 | 55 |  | 1.29×10^-5^ | 0.364 | 8.85×10^-10^ |
| 1.3810 | 1729 | 388 | 97 | 15157 | -470 | 89 | 171 |  | 6.61×10^-6^ | 1.090 | 4.40×10^-10^ |
| 1.3874 | 1826 | 444 | N/A | N/A | N/A | 229 | N/A |  | N/A | N/A | N/A |

**Fig. 3. (a) Confinement loss curves and (b) Amplitude Sensitivity curves of the proposed sensor.**

Fig. 3(b) displays the amplitude sensitivities for the listed RIs around their maximal values. Akin to *S_λ_*, similar physics lies behind the successively improved *S_A_* at higher wavelengths. The sensor sensitivity peaks at test RI 1.3810 (concentration = 380 mg/dL) to a magnitude of 470 RIU^-1^ in the amplitude interrogation as visualized in the figure. Sensor resolution is the measurement of the smallest quantifiable fluctuation in sample RI that can be detected or sensed by the device. The sensor resolution can be determined by the expression given below [48],

$R(RIU)=\frac{\Delta RI_{analyte}\times\Delta\lambda_{\min}}{\Delta\lambda_{res}}$ (6)

provided that the assumed minimal spectral resolution (*∆λ_min_*) is 0.1 nm which is standard for SPR-based technologies. The highest spectral resolution is obtained at the same point where the sensitivity was maximized (for RI=1.3810) which is 6.61×10^-6^ RIU. These performance parameters are tabulated in Table III as well for convenience. Moreover, Table IV gives a comparative impression on the performance of our work with recently reported SPR refractive index sensors for other bio-analyte and chemical detection applications.

Although a sensor's sensitivity can give a direct indication of the merit of detection and the degree of performance, there are additional factors to be considered for critically assessing a practicable sensor. Figure of merit (FOM) can be achieved as high as 171 RIU^-1^ for this sensor which results from the steep resonance loss peaks (narrow full-width half maxima, FWHM) exhibited by the leaky PCF. Both FOM and FWHM are illustrated graphically in Fig. 4(a) for our experimental sodium densities. The SPR wavelengths are fitted by a second order equation to observe the trend in sensing responses. The fitted curve is plotted against the analyte RIs in Fig 4(b), where we can see very good R^2^ (~unity) is found. The signal quality at resonant peaks and the minimum blood plasma sample required for viable operation can be determined by the signal-to-noise ratio (SNR) and limit of detection (DL) of the device. Our sensor demonstrated very promising SNRs and DLs for all of the cases as can be seen in the performance Table III (column 10-11). The proposed sensor exhibits the maximum SNR and DL values of 1.0899 and 1.32×10^-4^ RIU^2^/nm, respectively. Using the following two equations, SNR and DL of the sensor have been evaluated.

$SNR=\frac{\Delta\lambda_{res}}{FWHM}$ (7)

$DL=\frac{R_{\min}}{S_{\lambda}}$ (8)

**Fig. 4. (a) FOM & FWHM points and (b) Polynomial Fitting curve of the proposed sensor.**

##### **Optimization & tolerance analysis**

Structural parameters play crucial roles in fine-tuning the wave-guidance which ultimately influence the final sensing performance. Therefore, to obtain sensitivity-intensified performance, we examined the impact of dimensional variations by taking into account different PCF parameters. Our optimization gives estimations on sensor tolerances for possible imperfections that might appear after fabrication. The light penetration into a thin film and the excitation of the immobilized analyte ions are governed by the optical property and thickness of the plasmonic layer as well as the efficiency of the designated leakage pathways. Therefore, to begin with, we investigated different variations of the ITO layer’s depth (45 nm, 50 nm & 55 nm) at RIs 1.3674 and 1.3743, which are shown in Fig. 5. During this successive thickening of ITO layer, at sample RI 1.3674, we got the peak losses of 167 dB/cm, 203 dB/cm, and 251 dB/cm, respectively for the aforementioned thicknesses. For the RI of 1.3743, the sensor also shows the peak losses of 200 dB/cm, 257 dB/cm, and 347 dB/cm following the same order (see Fig. 5(a)). The wavelength sensitivity (*S_ʎ_*) has improved as the ITO layer’s thickness is increased which are 5072 nm/RIU, 6087 nm/RIU and 6957 nm/RIU due to the resonant wavelength shifts of 35 nm, 42 nm and 48 nm respectively. Similarly, in the case of amplitude sensitivity (*S_A_*), we got the peak values of 65 RIU^-1^, 99 RIU^-1^, and 152 RIU^-1^ correspondingly (see Fig. 5(b)). However, from the figure, it is evident that the values of the peak losses and *S_A_* are intensified with the increase of the depth of the plasmonic material. Here, we chose t=50 nm over 45 nm and 55 nm for the proposed sensor because of the loss profile consideration for practicability. For t=45 nm, though the sensor illustrates the lowest peak loss value, it has the lowest peak *S_A_* value. And for t=55 nm, our sensor exhibits an exact opposite trend for both loss and *S_A_*, where it shows the highest *S_A_* with very high loss magnitude. In kilometers-long communication fibers, only a few dB/cm loss is allowable for attaining sufficient energy output for signal reconstruction [50], however considering our optical setup (a few centimeters long fiber), the tolerable limit can be extended. Even so, this extension is not infinite by principle and that is why we had to sacrifice both *S_ʎ_* and *S_A_* to cap the loss within realizable threshold. Therefore, our selection of t=50 nm as the conducting layer is deemed the most suitable for our desired application.

**Fig. 5. (a) Loss spectrum and (b) Amplitude Sensitivity spectrum for varying ITO thickness in Y-polarized mode.**

Furthermore, the proposed sensor exhibited tolerable performance fluctuations in terms of its loss characteristics while examined for dimensional erosion and dilation up to ±10% from the optimized PCF parameters. The simulation result reveals the effects of the different diameters of the air holes and the pitch size variations for analyte RI 1.3743 which are displayed in Fig. 6. Modifying large air hole size does not cause any significant spectral position fluctuations. However, it regulates the peak losses to 174 dB/cm, 189 dB/cm, 218 dB/cm, and 232 dB/cm from the optimum value of 257 dB/cm when the optimized air-hole size (d=1.43 μm) was changed by +10%, +5%, -5%, and -10%, respectively (see Fig. 6(a)). In the case of scaled-down airholes, the sensor shows a slight change in loss characteristics again for ±10% variations in size. The maximum and minimum loss values of 214 dB/cm at 1645 nm and 194 dB/cm at 1626 nm were found for changes of +10% and -10% respectively from the proposed diameter (dc=0.44 μm) which are only +5.4% and -4.4% of the optimum value (see Fig. 6(b)). The wavelength shift of >20 nm compensates with the higher RI analytes and does not affect the performance in any way. Additionally, Figure 6(c) shows the spectrums of loss depth caused by varying pitch size. Loss peaks are gradually reduced when the pitch size is increased, and vice versa. In contrast, as a result of any reduction in pitch size, the RI contrast difference between core mode and SPP mode is elevated, which significantly raises loss depth resulting in an improved FOM. According to Fig. 6(c), if the optimum pitch size is modified by ±10%, the sensor illustrates peak losses of 145 dB/cm, 171 dB/cm, 245 dB/cm, and 295 dB/cm, respectively. Besides, the fluctuations were extremely small (fractions of nanometer) in terms of spectral shift. Simulation-wise, it is apparent that even if we modify all the suggested variables within a range from + 10% to -10%, its effect on the sensor's output remain trivial. Hence, if the loss depths and resonant peak wavelengths slightly change from their optimum values, the sensor can still ensure highly consistent operation.

**Fig. 6. Fabrication tolerance effects on (a) Large air hole variations, (b) Small air hole variations, and (c) Pitch size variations for Y-polarized mode.**

TABLE IV

Performance comparison of the proposed sensor with recently reported sensor

| Ref. | Structure Type | RI Range | Pol. Mode | Max.  Wave. Sens. | Max.  Wave. Reso. | Max.  Amp. Sens. |
| --- | --- | --- | --- | --- | --- | --- |
| [20] | Titanium Nitride Coated PCF-SPR Sensor | 1.385–1.40 | Y-pol | 10,000 | 2.0×10^-5^ | 70 |
| [21] | SPR-PCF Sensor For Early-Stage Cancer Detection | 1.3246–1.3634 | X-pol Y-pol | 6701.03 5154.63 | -  - | 110  - |
| [22] | MXene-coated PCF for Malaria Detection in RBCs | 1.36-1.40 | X-pol | 12,142 | 1.21×10^-5^ | - |
| [23] | PCF Biosensor for SARS-CoV-2 Particles | 1.3348-1.3576 | Y-pol | 3,948 | 2.53×10^-5^ | 240 |
| [24] | U-channel SPR-PCF for Hemoglobin Sensing | 1.26-1.42 | Y-pol | 7,500 | 6.0×10^-3^ | - |
| [28] | D-shaped PCF Sensor with Graphene and ZnO | 1.36–1.41 | Y-pol | 4,486 | 1.667×10^-5^ | - |
| Proposed sensor | Testing of Hyponatremia and Hypernatremia Level Sensor | 1.3602-1.3874 | Y-pol | 15,157 | 2.0×10^-5^ | 470 |

##### **Conclusion**

In summary, the proposed SPR-PCF sensor is developed for rapid serum sodium electrolyte detection that shows highly optically sensitive performance. We numerically analyzed the detection performance of our SPR-PCF sensor to confirm the best sensing operation. The sensor resolution spans from 6.6× 10^-6^ RIU to 2.0× 10^-5^ RIU with the maximum wavelength and amplitude sensitivity of 15,160 nm/RIU and 470 RIU^-1^, respectively. In the FEM-based simulations, we validated the structural tolerances are within practical limits. Additionally, it shows a high birefringence peak of approximately 11.4 × 10^-4^. We anticipate that the proposed polarization-sensitive sensor will be an effective POC tool for hypo- or hypernatremia fast identification in both at-home and nosocomial circumstances.

**Supporting Information**

All data underlying the results presented in this paper and necessary to replicate our study findings are available from the COMSOL Multiphysics file (S_sens_ito.m file) that is zipped and attached as the supporting information.

##### **Acknowledgment**

A.M.T.H and A.I authors contributed equally to this work.

##### **References**

[1]	C. Svensen, “42 - Electrolytes and Diuretics,” in *Pharmacology and Physiology for Anesthesia (Second Edition)*, H. C. Hemmings and T. D. Egan, Eds., Philadelphia: Elsevier, 2019, pp. 814–835. doi: 10.1016/B978-0-323-48110-6.00042-9.

[2]	S. Gadewar and A. Fasano, “Current concepts in the evaluation, diagnosis and management of acute infectious diarrhea,” *Curr. Opin. Pharmacol.*, vol. 5, no. 6, pp. 559–565, Dec. 2005, doi: 10.1016/j.coph.2005.08.002.

[3]	“Evaluation of Electrolytes Imbalance and Dyslipidemia in Diabetic Patients.” Accessed: Jan. 20, 2025. [Online]. Available: https://www.mdpi.com/2076-3271/4/2/7

[4]	“Molecular and physical technologies for monitoring fluid and electrolyte imbalance: A focus on cancer population - Bennet - 2021 - Clinical and Translational Medicine - Wiley Online Library.” Accessed: Jan. 20, 2025. [Online]. Available: https://onlinelibrary.wiley.com/doi/full/10.1002/ctm2.461

[5]	“Fluid and Electrolyte Disturbances in COVID‐19 and Their Complications - Pourfridoni - 2021 - BioMed Research International - Wiley Online Library.” Accessed: Jan. 20, 2025. [Online]. Available: https://onlinelibrary.wiley.com/doi/full/10.1155/2021/6667047

[6]	Y.-H. Chou *et al.*, “Restoration of dysnatremia and acute kidney injury benefits outcomes of acute geriatric inpatients,” *Sci. Rep.*, vol. 11, no. 1, p. 20097, Oct. 2021, doi: 10.1038/s41598-021-99677-z.

[7]	“Dysnatremia and risk of bloodstream infection in dialysis patients | Clinical Kidney Journal | Oxford Academic.” Accessed: Jan. 20, 2025. [Online]. Available: https://academic.oup.com/ckj/article/15/12/2322/6694006

[8]	“Management of hyponatremia | CMAJ.” Accessed: Jan. 20, 2025. [Online]. Available: https://www.cmaj.ca/content/186/8/E281.short

[9]	S. A. Muhsin and D. B. Mount, “Diagnosis and treatment of hypernatremia,” *Best Pract. Res. Clin. Endocrinol. Metab.*, vol. 30, no. 2, pp. 189–203, Mar. 2016, doi: 10.1016/j.beem.2016.02.014.

[10]	“The Effect of Electrolytes on Blood Pressure: A Brief Summary of Meta-Analyses.” Accessed: Jan. 20, 2025. [Online]. Available: https://www.mdpi.com/2072-6643/11/6/1362

[11]	R. M. Reynolds, P. L. Padfield, and J. R. Seckl, “Disorders of sodium balance,” *BMJ*, vol. 332, no. 7543, pp. 702–705, Mar. 2006, doi: 10.1136/bmj.332.7543.702.

[12]	W. L. Wright, “Sodium and Fluid Management in Acute Brain Injury,” *Curr. Neurol. Neurosci. Rep.*, vol. 12, no. 4, pp. 466–473, Aug. 2012, doi: 10.1007/s11910-012-0284-5.

[13]	E. Salman, A. Kadota, and K. Miura, “Global guidelines recommendations for dietary sodium and potassium intake,” *Hypertens. Res.*, vol. 47, no. 6, pp. 1620–1626, Jun. 2024, doi: 10.1038/s41440-024-01663-1.

[14]	B. Lee and H.-D. Park, “Performance evaluation of the i-Smart 300E cartridge for point-of-care electrolyte measurement in serum and plasma,” *J. Clin. Lab. Anal.*, vol. 36, no. 4, p. e24295, 2022, doi: 10.1002/jcla.24295.

[15]	H. J. Adrogué and N. E. Madias, “Hypernatremia,” *N. Engl. J. Med.*, vol. 342, no. 20, pp. 1493–1499, May 2000, doi: 10.1056/NEJM200005183422006.

[16]	A. Baruah, P. Goyal, S. Sinha, K. L. Ramesh, and R. Datta, “Delay in Specimen Processing-Major Source of Preanalytical Variation in Serum Electrolytes,” *J. Clin. Diagn. Res. JCDR*, vol. 8, no. 12, pp. CC01–CC03, Dec. 2014, doi: 10.7860/JCDR/2014/10150.5285.

[17]	“Wilderness Medical Society Practice Guidelines for Treatment of Exercise-Associated Hyponatremia - Brad L. Bennett, Tamara Hew-Butler, Martin D. Hoffman, Ian R. Rogers, Mitchell H. Rosner, 2013.” Accessed: Jan. 20, 2025. [Online]. Available: https://journals.sagepub.com/doi/full/10.1016/j.wem.2013.01.011

[18]	J. Kwon *et al.*, “Artificial intelligence for detecting electrolyte imbalance using electrocardiography,” *Ann. Noninvasive Electrocardiol.*, vol. 26, no. 3, p. e12839, 2021, doi: 10.1111/anec.12839.

[19]	“Comparison of the point-of-care blood gas analyzer versus the laboratory auto-analyzer for the measurement of electrolytes | International Journal of Emergency Medicine.” Accessed: Jan. 20, 2025. [Online]. Available: https://link.springer.com/article/10.1007/s12245-009-0091-1

[20]	C.-T. C. Chao, M. R. R. Kooh, Y.-F. C. Chau, and R. Thotagamuge, “Susceptible Plasmonic Photonic Crystal Fiber Sensor with Elliptical Air Holes and External-Flat Gold-Coated Surface,” *Photonics*, vol. 9, no. 12, Art. no. 12, Dec. 2022, doi: 10.3390/photonics9120916.

[21]	C.-T. C. Chao *et al.*, “Improving Temperature-Sensing Performance of Photonic Crystal Fiber via External Metal-Coated Trapezoidal-Shaped Surface,” *Crystals*, vol. 13, no. 5, Art. no. 5, May 2023, doi: 10.3390/cryst13050813.

[22]	Y.-F. C. Chau *et al.*, “Improved Refractive Index-Sensing Performance of Multimode Fano-Resonance-Based Metal-Insulator-Metal Nanostructures,” *Nanomaterials*, vol. 11, no. 8, Art. no. 8, Aug. 2021, doi: 10.3390/nano11082097.

[23]	N. R. Sabaruddin, Y. M. Tan, C.-T. Chou Chao, M. R. R. Kooh, and Y.-F. Chou Chau, “High Sensitivity of Metasurface-Based Five-Band Terahertz Absorber,” *Plasmonics*, vol. 19, no. 1, pp. 481–493, Feb. 2024, doi: 10.1007/s11468-023-01989-5.

[24]	C.-T. C. Chao, M. R. R. Kooh, C. M. Lim, R. Thotagamuge, A. H. Mahadi, and Y.-F. C. Chau, “Visible-Range Multiple-Channel Metal-Shell Rod-Shaped Narrowband Plasmonic Metamaterial Absorber for Refractive Index and Temperature Sensing,” *Micromachines*, vol. 14, no. 2, Art. no. 2, Feb. 2023, doi: 10.3390/mi14020340.

[25]	S. Jiao, S. Gu, H. Fang, and H. Yang, “Analysis of Dual-Core Photonic Crystal Fiber Based on Surface Plasmon Resonance Sensor with Segmented Silver Film,” *Plasmonics*, vol. 14, no. 3, pp. 685–693, Jun. 2019, doi: 10.1007/s11468-018-0846-8.

[26]	V. S. Chaudhary, D. Kumar, B. P. Pandey, and S. Kumar, “Advances in Photonic Crystal Fiber-Based Sensor for Detection of Physical and Biochemical Parameters—A Review,” *IEEE Sens. J.*, vol. 23, no. 2, pp. 1012–1023, Jan. 2023, doi: 10.1109/JSEN.2022.3222969.

[27]	V. Kaur and S. Singh, “Design of titanium nitride coated PCF-SPR sensor for liquid sensing applications,” *Opt. Fiber Technol.*, vol. 48, pp. 159–164, Mar. 2019, doi: 10.1016/j.yofte.2018.12.015.

[28]	D. Rajeswari and A. A. Revathi, “Highly sensitive SPR-based PCF bio sensor for plasma cell detection in human blood for the detection of early stage cancer,” *Optik*, vol. 258, p. 168897, May 2022, doi: 10.1016/j.ijleo.2022.168897.

[29]	A. Kumar, P. Verma, and P. Jindal, “Machine learning approach to surface plasmon resonance sensor based on MXene coated PCF for malaria disease detection in RBCs,” *Optik*, vol. 274, p. 170549, Mar. 2023, doi: 10.1016/j.ijleo.2023.170549.

[30]	A. Islam, F. Haider, R. A. Aoni, and R. Ahmed, “Plasmonic photonic biosensor: in situ detection and quantification of SARS-CoV-2 particles,” *Opt. Express*, vol. 30, no. 22, pp. 40277–40291, Oct. 2022, doi: 10.1364/OE.469937.

[31]	T. Dai *et al.*, “Ultra-high sensitivity surface plasmon U-channel photonic crystal fiber for hemoglobin sensing,” *Sens. Actuators Phys.*, vol. 366, p. 115053, Feb. 2024, doi: 10.1016/j.sna.2024.115053.

[32]	J.-F. Masson, “Portable and field-deployed surface plasmon resonance and plasmonic sensors,” *Analyst*, vol. 145, no. 11, pp. 3776–3800, Jun. 2020, doi: 10.1039/D0AN00316F.

[33]	F. Haider, R. A. Aoni, R. Ahmed, W. J. Chew, and G. A. Mahdiraji, “Alphabetic-Core Assisted Microstructure Fiber Based Plasmonic Biosensor,” *Plasmonics*, vol. 15, no. 6, pp. 1949–1958, Dec. 2020, doi: 10.1007/s11468-020-01220-9.

[34]	F. Haider, R. A. Aoni, R. Ahmed, Md. S. Islam, and A. E. Miroshnichenko, “Propagation Controlled Photonic Crystal Fiber-Based Plasmonic Sensor via Scaled-Down Approach,” *IEEE Sens. J.*, vol. 19, no. 3, pp. 962–969, Feb. 2019, doi: 10.1109/JSEN.2018.2880161.

[35]	D. Pysz *et al.*, “Stack and draw fabrication of soft glass microstructured fiber optics,” *Pol. Akad. Nauk Bull. Pol. Acad. Sci. Tech. Sci.*, vol. 62, no. 4, pp. 667–682, Dec. 2014, doi: 10.2478/bpasts-2014-0073.

[36]	M. Kiroriwal, P. Singal, M. Sharma, and A. Singal, “Hemoglobin sensor based on external gold-coated photonic crystal fiber,” *Opt. Laser Technol.*, vol. 149, p. 107817, May 2022, doi: 10.1016/j.optlastec.2021.107817.

[37]	A. K. Shakya and S. Singh, “Design of a novel refractive index BIOSENSOR for heavy metal detection from water samples based on fusion of spectroscopy and refractive index sensing,” *Optik*, vol. 270, p. 169892, Nov. 2022, doi: 10.1016/j.ijleo.2022.169892.

[38]	A. K. Shakya and S. Singh, “State of the Art Alliance of Refractive Index Sensing and Spectroscopy Techniques for Household Oils Analysis,” *Plasmonics*, vol. 18, no. 6, pp. 2347–2364, Dec. 2023, doi: 10.1007/s11468-023-01940-8.

[39]	A. Kumar Shakya and S. Singh, “Design of novel Penta core PCF SPR RI sensor based on fusion of IMD and EMD techniques for analysis of water and transformer oil,” *Measurement*, vol. 188, p. 110513, Jan. 2022, doi: 10.1016/j.measurement.2021.110513.

[40]	“Highly Sensitive Surface Plasmon Resonance Refractive Index Sensor Based on D-Shaped Dual‑Core Photonic Crystal Fiber with ITO Film | Plasmonics.” Accessed: Jan. 20, 2025. [Online]. Available: https://link.springer.com/article/10.1007/s11468-023-02103-5

[41]	“A D-Shaped Photonic Crystal Fiber Refractive Index Sensor Coated with Graphene and Zinc Oxide.” Accessed: Jan. 20, 2025. [Online]. Available: https://www.mdpi.com/1424-8220/21/1/71

[42]	“High sensitivity photonic crystal fiber surface plasmon resonance sensor based on indium tin oxide | Optical and Quantum Electronics.” Accessed: Jan. 20, 2025. [Online]. Available: https://link.springer.com/article/10.1007/s11082-023-04890-x

[43]	“Point of Care Detection of Blood Electrolytes and Glucose Utilizing Nano-Dot Enhanced Plasmonic Biosensor | IEEE Journals & Magazine | IEEE Xplore.” Accessed: Jan. 20, 2025. [Online]. Available: https://ieeexplore.ieee.org/abstract/document/9438684

[44]	“Immobilization of Sodium Ions on the Pore Surface of a Porous Coordination Polymer | Journal of the American Chemical Society.” Accessed: Jan. 20, 2025. [Online]. Available: https://pubs.acs.org/doi/full/10.1021/ja0606879

[45]	J. Yang *et al.*, “Na and Cl immobilization by size controlled calcium silicate hydrate nanometer pores,” *Constr. Build. Mater.*, vol. 202, pp. 622–635, Mar. 2019, doi: 10.1016/j.conbuildmat.2019.01.066.

[46]	“U-Grooved Selectively Coated and Highly Sensitive PCF-SPR Sensor for Broad Range Analyte RI Detection | IEEE Journals & Magazine | IEEE Xplore.” Accessed: Jan. 20, 2025. [Online]. Available: https://ieeexplore.ieee.org/abstract/document/10080952

[47]	Q. Cheng, S. Fan, Y. Guan, J. Dong, and G. Xu, “Enhanced sensitivity of temperature sensor by a novel birefringent photonic crystal fiber with PDMS filling,” *Opt. Commun.*, vol. 565, p. 130697, Aug. 2024, doi: 10.1016/j.optcom.2024.130697.

[48]	A. M. T. Hoque, A. Islam, F. Haider, H. A. B. A. Rashid, R. Ahmed, and R. A. Aoni, “Dual polarized surface plasmon resonance refractive index sensor via decentering propagation-controlled core sensor,” *Opt. Contin.*, vol. 1, no. 7, pp. 1474–1488, Jul. 2022, doi: 10.1364/OPTCON.460520.

[49]	“Photonic crystal fiber refractive index sensor based on SPR | Optical and Quantum Electronics.” Accessed: Jan. 20, 2025. [Online]. Available: https://link.springer.com/article/10.1007/s11082-022-04324-0

[50]	A. A. S. Falah, W. R. Wong, and F. R. Mahamd Adikan, “Single-mode eccentric-core D-shaped photonic crystal fiber surface plasmon resonance sensor,” *Opt. Laser Technol.*, vol. 145, p. 107474, Jan. 2022, doi: 10.1016/j.optlastec.2021.107474.

1. ^ ^
