# Supplementary material for: Point-of-care testing of hyponatremia and hypernatremia levels: An optoplasmonic biosensing approach
Source: PLoS One. 2025 Apr 23;20(4):e0319559. doi: 10.1371/journal.pone.0319559 (PMC12017493; doi:10.1371/journal.pone.0319559)
Supplement: Attachment — Submitted filename: Comment Sheet.docx [file pone.0319559.s002.docx]

***Article:* Point-of-care testing of hyponatremia and hypernatremia levels: an optoplasmonic biosensing approach**

**Comments to Author**

1. How does the imbalance of Na+ ions in blood or lymph impact a patient’s health and why is it crucial to monitor sodium ion (Na+) levels in the human body properly explain the mecial cause?
2. What limitations in current point-of-care (POC) testing for Na+ levels does this study aim to address and do photonic crystal fiber (PCF)-based surface plasmon resonance (SPR) sensor detect those shortcomings?
3. How does the modified square lattice design improve the waveguidance and sensing performance of the proposed PCF sensor
4. Why was ITO chosen as the plasmonically active material, over other materail like Au, Ag and how does it enhance the sensor's performance?
5. How does structural asymmetry-induced birefringence contribute to the superior sensing response in the y-polarized mode compared to the x-polarized mode?
6. What does a sensitivity of 15,157 nm/RIU indicate about the sensor's performance or this is just a numerical figure only?
7. What is the significance of achieving a detection resolution in the order of 10^−6^ RIU in practical applications?
8. How does the proposed sensor compare to conventional serum electrolyte panels currently used in medical diagnostics?
9. In what ways could this sensor be utilized as a POC alternative for rapid Na+ ion testing?
10. Update the introduction section of the article with some latest models of PCF SPR sensor like mereger of IMD and EMD shaped model, EMD and D shaped model, meger of spectroscopy and RI sensing etc. Cite them in the intoduction section?

https://doi.org/10.1007/s11468-023-01940-8

https://doi.org/10.1016/j.measurement.2021.110513

https://doi.org/10.1016/j.ijleo.2022.169892

**Comments to Editor**

Dear Sir, Good Morning

Authors have done good work in the article. I recommend acceptance of the article with**"minor revison".**
